# A novel chondrocyte sheet fabrication using human-induced pluripotent stem cell-derived expandable limb-bud mesenchymal cells

**DOI:** 10.1186/s13287-023-03252-4

**Published:** 2023-02-24

**Authors:** Tomoka Takao, Masato Sato, Yuki Fujisawa, Eriko Toyoda, Daisuke Yamada, Yukio Hitsumoto, Eiji Nakata, Toshifumi Ozaki, Takeshi Takarada

**Affiliations:** 1 Department of Regenerative Science, Dentistry and Pharmaceutical Sciences, Okayama University Graduate School of Medicine, 2-5-1 Shikata-Cho, Kita-Ku, Okayama, 700-8558 Japan; 2 Department of Orthopaedic Surgery, Surgical Science, Tokai University School of Medicine, Isehara, Japan; 3 Department Orthopedic Surgery, Dentistry and Pharmaceutical Sciences, Okayama University Graduate School of Medicine, Okayama, 700-8558 Japan

**Keywords:** Chondrocyte sheet, Human-induced pluripotent stem cells, Expandable limb-bud mesenchymal cells

## Abstract

**Background:**

Cell sheet fabrication for articular cartilage regenerative medicine necessitates a large number of chondrocytes of consistent quality as a cell source. Previously, we have developed human-induced pluripotent stem cell (iPSC)-derived expandable PRRX1^+^ limb-bud mesenchymal cells (ExpLBM) with stable expansion and high chondrogenic capacity, while in this study; our ExpLBM technology was combined with cell sheet engineering to assess its potential as a stable cell source for articular cartilage regeneration.

**Methods:**

ExpLBM cells derived from human-induced pluripotent stem cells (hiPSCs), including 414C2 and Ff-KVs09 (HLA homozygous), were seeded onto a culture plate and two-dimensional chondrogenic induction (2-DCI) was initiated. After 2-DCI, ExpLBM-derived chondrocytes were stripped and transferred to temperature-responsive culture inserts and the chondrocyte sheets were histologically examined or transplanted into osteochondral knee defects of immunodeficient rats.

**Results:**

Immunohistochemistry revealed that ExpLBM-derived cell sheets were positive for Safranin O, COL2, and ACAN but that they were negative for COL1 and RUNX2. Furthermore, the engrafted tissues in osteochondral knee defects in immunodeficient rats were stained with SafO, human VIMENTIN, ACAN, and COL2.

**Conclusions:**

The present study is the first to report the chondrocyte sheet fabrication with hiPSC-derived cell source. hiPSC-derived ExpLBM would be a promising cell source for cell sheet technology in articular cartilage regenerative medicine.

**Supplementary Information:**

The online version contains supplementary material available at 10.1186/s13287-023-03252-4.

## Background

Articular cartilage is made up of chondrocytes that are seen to be randomly distributed and embedded in an extracellular matrix (ECM) that is mostly made up of Type II collagen and proteoglycans. This ECM absorbs external shock and facilitates smooth articular movements [1, 2]. We see therefore that spontaneous regeneration of their defects caused by traumatic injury or osteoarthritis (OA) is difficult due to the avascular structure of articular cartilage. Where chondrocyte transplantation derived from auto- or allografts is one of the therapies used to prevent or delay OA progression [3, 4], focal cartilage defects have thus been identified as a potential risk factor for OA. Current graft approaches for small cartilage defects include osteochondral autograft transfer [5], osteochondral allograft transplantation [6], and autologous chondrocyte implantation [7, 8], however, their need for a large number of chondrocytes or limited applications restrict graft options.

Recently, chondrocyte sheets have been engineered as a therapeutic strategy for articular cartilage defects. While Sato et al. have conducted a clinical study to test the autologous transplantation of human chondrocyte sheets in knee defects of patients with OA and confirmed no serious adverse events after more than 3 years [9], it is seen that chondrocytes proliferate on temperature-responsive culture devices such as thermo-responsive polymer-coated culture dishes or devices [10, 11], forming sheets that can be collected by lowering the temperature. In addition, the safety and therapeutic effect of chondrocyte sheets have been demonstrated in several animal models [12–15].

Previously, we demonstrated that human-induced pluripotent stem cell (hiPSC)-derived expandable limb-bud mesenchymal cells (ExpLBM) are stably expandable while maintaining high chondrogenic capacity in xeno-free culture conditions [16]. Human cartilage tissues in the cranial, axial, and appendicular skeletons are ontogenically generated from the neural crest, paraxial mesoderm, and lateral plate mesoderm-derived lineages, respectively [17–19], which implies that lateral plate mesoderm-derived ExpLBM would be a desirable cell source for the regeneration of limb articular cartilages. In this study, ExpLBM was found to produce functional and engraftable chondrocyte sheets when we combined the ExpLBM with cell sheet technology.

## Materials and methods

Complete materials and methods are presented in Additional file 1: Supplemental materials and methods.

## Results

**Fig. 1:**
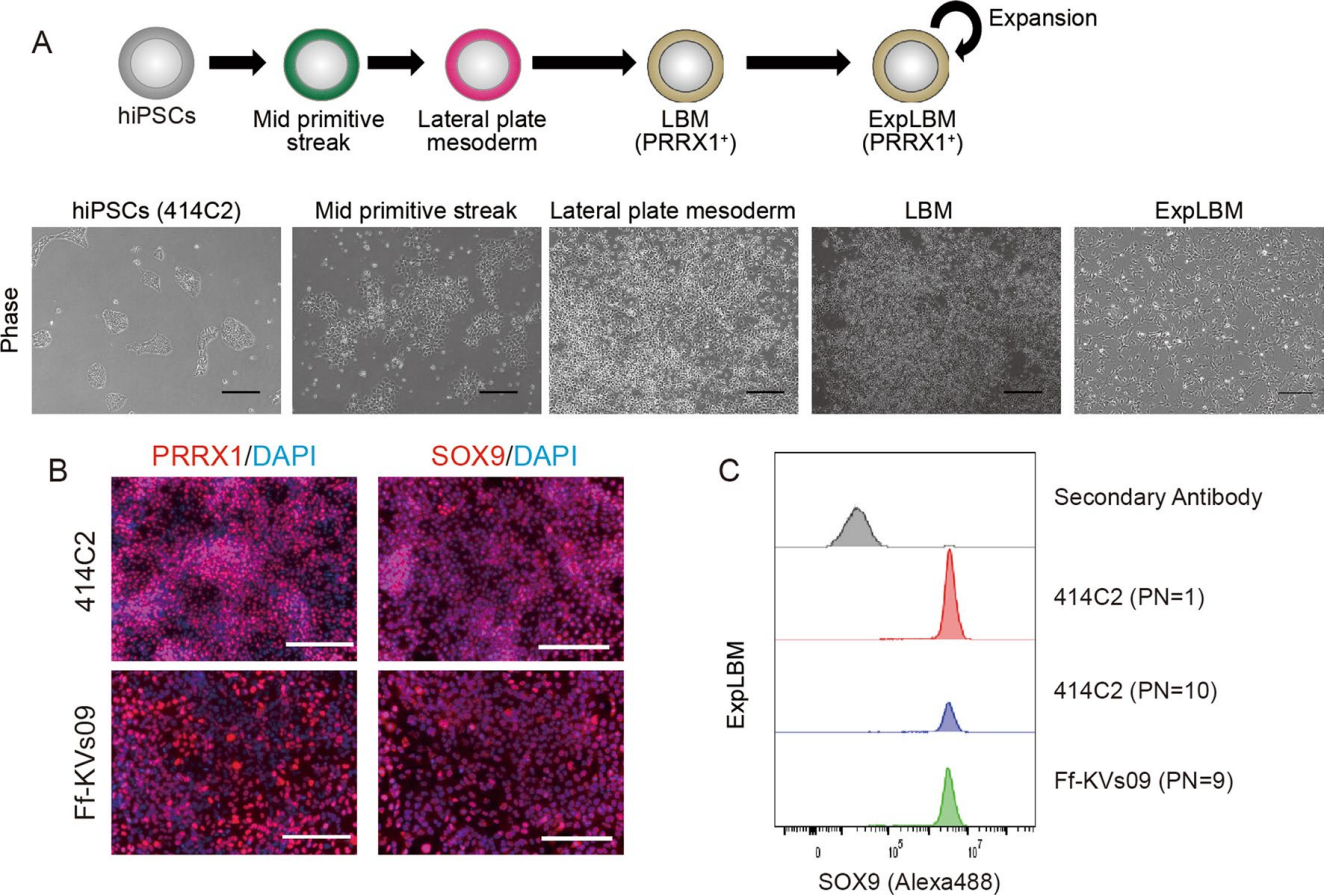
Induction of ontogenetically defined ExpLBM from hiPSCs. **A** A step-by-step differentiation procedure for inducing ExpLBM from hiPSCs. Each phase photograph was taken at the specified time points. Representative images are displayed (scale bar: 200 µm). **B** Immunostaining for PRRX1 and SOX9 in 414C2 or HLA-homozygous Ff-KVs09-derived ExpLBM (scale bar: 200 µm). **C** Flow cytometric analysis of SOX9 expression in 414C2 or Ff-KVs09-derived ExpLBM cells at each indicated passage number (PN)

Previously, we ontogenically induced limb-bud mesenchyme (LBM) from hiPSCs and established their expansion method [16]. Figure 1A depicts the cellular morphology of 414C2 hiPSCs at each stage of differentiation. Due to the fact that immune rejection is one of the most serious problems in regenerative medicine [20], HLA-homozygous hiPSCs (Ff-KVs09) were also tested in this study. ExpLBM cells derived from 414C2 hiPSCs (414C2 ExpLBM) and Ff-KVs09 HLA-homozygous hiPSCs (Ff-KVs09 ExpLBM) expressed PRRX1 and SOX9 stably (Fig. 1B), and maintained an almost 100% positive rate of SOX9 expression during serial passage (Fig. 1C).

**Fig. 2:**
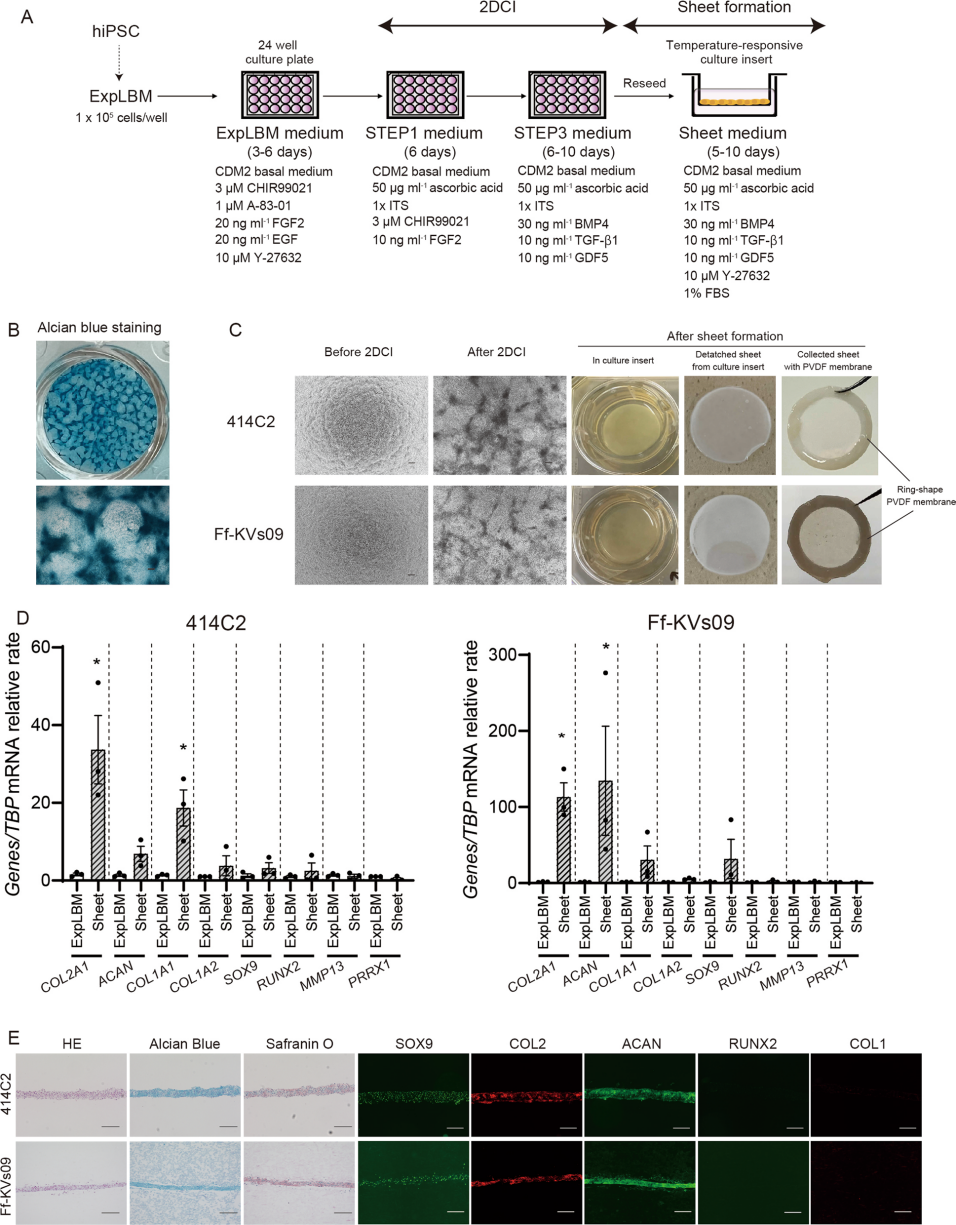
Fabrication and characterization of ExpLBM-derived chondrocyte sheets. **A** A diagram of ExpLBM-derived chondrocyte sheet fabrication. At the end of 2-DCI, ExpLBM-derived chondrocytes were dissociated and seeded onto a temperature-responsive culture insert. **B** Alcian blue staining after 2-DCI (scale bar: 200 µm). **C** Fabrication of 414C2 or Ff-KVs09 ExpLBM-derived sheet-like structures. Images were acquired at the specified time points (scale bar: 200 µm). **D** Quantitative reverse-transcription-polymerase chain reaction (qRT-PCR) analysis of chondrocyte marker genes in ExpLBM-derived chondrocyte sheets. Total RNAs were extracted, and the expression levels of each gene were compared between ExpLBM and chondrocyte sheets. All values were normalized to *TBP* mRNA levels (*n* = 3, three biologically independent experiments). **E** Histological examination of 414C2 or Ff-KVs09 ExpLBM-derived chondrocyte sheets. Chondrocyte sheet sections were stained with hematoxylin and eosin (H&E), Alcian blue, or Safranin O, or with antibodies against SOX9, COL2, ACAN, COL1, and RUNX2 (scale bars: 200 µm)

To fabricate the ExpLBM-derived chondrocyte sheet, ExpLBM cells were first used in a two-dimensional (adhesive culture) chondrogenic induction (2-DCI) protocol with STEP1 and STEP3 medium for inducing an ExpLBM-derived chondrocyte sheet (Fig. 2A). After chondrocyte induction using the 2-DCI protocol, 414C2 ExpLBM produced several nodules stained with Alcian blue (Fig. 2B). Following 2-DCI, ExpLBM-derived chondrocytes were dissociated, reseeded on temperature-responsive culture inserts, and cultured in a sheet medium. ExpLBM-derived chondrocyte sheets with high cell density formed a thick structure with an integrated layer that was easily harvested and manipulated without tearing (Fig. 2C). RT-qPCR analysis revealed that *COL2A1* and *COL1A1* were significantly upregulated in 414C2 ExpLBM-derived chondrocyte sheets rather than ExpLBM cells, whereas Ff-KVs09 ExpLBM-derived chondrocytes showed significantly higher expression of *COL2A1* and *ACAN* compared to Ff-KVs09 ExpLBM cells (Fig. 2D). Histological examination of 414C2 and Ff-KVs09 ExpLBM-derived chondrocyte sheets revealed that the cells were embedded in ECM that was intensely and homogeneously stained with Alcian blue and Safranin O. Immunohistochemistry revealed that all cells used were embedded in the ECM expressed SOX9 but not RUNX2; Type II collagen (COL2) was detected in the ECM but not COL1 (Fig. 2E). Although we found some differences among hiPSC clones in cellular characteristics of chondrocyte sheets, these findings suggest that ExpLBM-derived chondrocyte sheets exhibit hyaline cartilage-like characteristics.

**Fig. 3:**
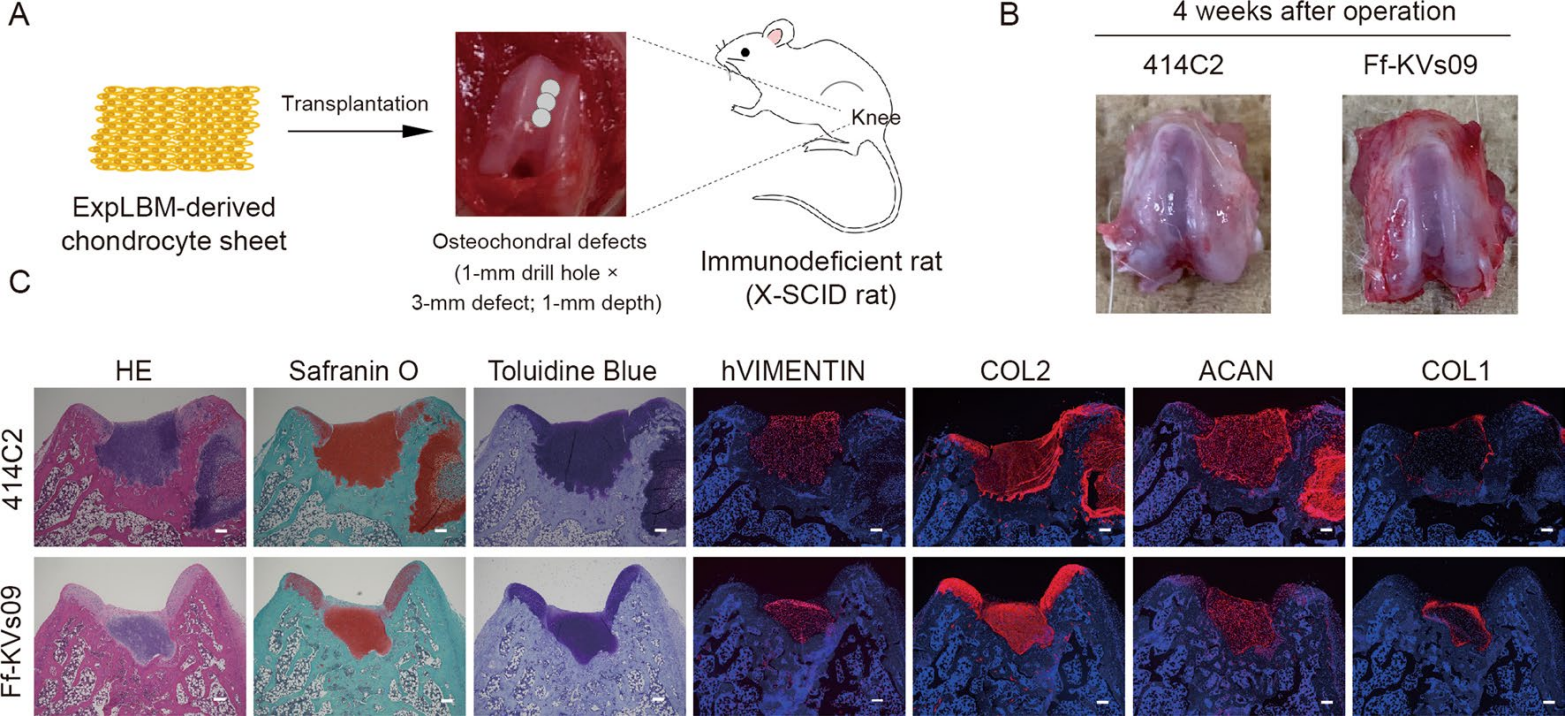
Engraftment of ExpLBM-derived chondrocyte sheets in X-SCID rats. **A** A diagram of ExpLBM-derived chondrocyte sheet transplantation procedures. **B** The knee joints four weeks after transplantation. **C** Histological examination of osteochondral defects that have been engrafted with ExpLBM-derived chondrocyte sheets. Four weeks after transplantation, the knee joints were fixed, and tissue sections were stained with H&E, Safranin O, or toluidine blue, or antibodies against human VIMENTIN, COL2, ACAN, or COL1. Representative images are displayed (scale bars: 200 µm)

To demonstrate regenerative efficacy in vivo, these ExpLBM-derived chondrocyte sheets were transplanted into osteochondral defects (1-mm drill hole × 3 defects; 1-mm depth) created in the knee joint cartilage of X-linked severe combined immunodeficiency (X-SCID) rats (Fig. 3A). Four weeks after transplantation, both 414C2 and Ff-KVs09 ExpLBM-derived chondrocyte sheets were successfully engrafted into osteochondral defects. The defects were filled with ExpLBM-derived chondrocytes, as evidenced by human vimentin (hVIMENTIN) expression. These chondrocyte sheets produced ECM, which was stained with toluidine blue and Safranin O. Immunohistochemistry revealed that COL2 and ACAN were found in numerous ECM regions of regenerated neocartilage, but COL1 was found only in the neocartilage surfaces (Fig. 3B and C). However, there were two limitations to this study. First, 4 weeks post transplantation is a short period for evaluating cartilage repair. Second, this study was conducted on immune-deficient rats. The validation of efficacy of transplantation is required in larger animals that more closely resemble humans. We plan to verify the efficacy of various cell source for ExpLBM-induced chondrocyte sheets for long time transplantation using large animals.

## Conclusions

In this study, we successfully fabricated an ExpLBM-derived chondrocyte sheet positive for COL2 and ACAN, which can be used as a potential regenerative resource for hyaline cartilage tissues. To our knowledge, this present study is the first to report the chondrocyte sheet fabrication with hiPSC-derived cell source. Given the challenges of human cartilage-derived chondrocyte cell sources with regard to their stable expansion while chondrogenic activity is maintained, our cell source of hiPSC-derived ExpLBM has the potential to stably provide a sufficient number of human chondrocytes for cell sheet fabrication in regenerative medicine. Furthermore, where we see that HLA-homozygous hiPSC-derived ExpLBM can reduce the risk of immune rejection due to HLA mismatching, taken together, hiPSC-derived ExpLBM would be a promising cell source for cell sheet technology in articular cartilage regenerative medicine.

## Supplementary Information

**Additional file 1.** Supplemental materials and methods.

## Abbreviations

ExpLBM: Expandable PRRX1^+^ LBM.
hiPSCs: Human-induced pluripotent stem cells.
2-DCI: Two-dimensional chondrogenic induction.
ECM: Extracellular matrix.
OA: Osteoarthritis.
LBM: Limb-bud mesenchyme

## Declarations

### Availability of data and materials

All data generated or analyzed during this study are included in this article.
