# Supplementary material for: A novel chondrocyte sheet fabrication using human-induced pluripotent stem cell-derived expandable limb-bud mesenchymal cells
Source: Stem Cell Res Ther. 2023 Feb 24;14:34. doi: 10.1186/s13287-023-03252-4 (PMC9960196; doi:10.1186/s13287-023-03252-4)
Supplement: Supplementary file 1 — Additional file 1. Supplemental materials and methods. [file 13287_2023_3252_MOESM1_ESM.docx]

Additional File1.

**Materials and methods**

**Cell culture:** HiPSCs were maintained and cultured using StemFit (AK02N, Ajinomoto). Before reaching subconfluence, cells were dissociated with TrypLE Select (Thermo Fisher Scientific)/0.25 mM EDTA and suspended in StemFit containing 10 μM Y27632 (FUJIFILM Wako). HiPSCs (1 × 10^4^) were suspended in StemFit with 10 μM of Y27632 and 8 μL of iMatrix511-silk (human laminin-511 E8 fragment, Nippi) and placed in a 6-cm dish. With the culture media being changed every two days until the next passage, the next day, following the suspension, the media were replaced with fresh StemFit without Y27632. The 414C2 hiPSCs or HLA-homozygous Ff-KVs09 hiPSCs were provided by the Center for iPS Cell Research and Application, Kyoto University.

For step-wise differentiation into limb-bud mesenchymal cells (LBM), hiPSCs (3 × 10^4^) were suspended in 1 mL of StemFit with 10 µM of Y27632, and 4 µL of iMatrix511-silk were added to a 3.5-cm culture dish. The next day, the culture medium was replaced with fresh StemFit without Y27632, and hiPSCs were differentiated, as previously described, into a midprimitive streak, lateral plate mesoderm, and LBM cells [16].

For serial passages of ExpLBM cells, LBM cells were dissociated using accutase (Thermo Fisher Scientific), and 2–4 × 10^5^ cells were suspended in ExpLBM medium (CDM2 basal medium + 3 μM of CHIR99021 + 1 μM of A-83-01 + 20 ng/mL of fibroblast growth factor 2 + 20 ng/mL of epidermal growth factor + 10 μM of Y27632) and cultured on a 6-cm dish coated with 4 μg/mL of human plasma fibronectin (Merck). The culture media was replaced with a fresh ExpLBM medium every 2 days. Before reaching subconfluence, the cells were passaged as described above.

**Immunocytochemistry:** Cells cultured on dishes were fixed with 4% paraformaldehyde for 30 minutes at room temperature and then incubated with blocking solution for 1 hour at room temperature [3% normal goat serum and 0.1% Triton X-100 in phosphate-buffered saline (PBS)]. The primary or secondary antibodies were diluted 200-or 500-fold in blocking solution and added to the cell cultures for 1 hour at room temperature. After incubation, the nuclei were stained with 0.1 μg/ml of 4′,6-diamidino-2-phenylindole (DAPI; Thermo Fisher). The samples were examined using a BZ-X710 fluorescence microscope (Keyence). The primary antibodies used were anti-PRRX1 (catalog no. ZRB2165; Sigma-Aldrich) and anti-SOX9 (catalog no. AB5535; Merck).

**Flow cytometry:** ExpLBM cells (1 × 10^5^) were suspended in 100 μl of 2% fetal bovine serum in PBS and stained with SOX9 antibody (diluted 1:200, AB5535, Merck) using a transcription factor buffer set (BD Biosciences), following the manufacturer’s instructions. After staining with antirabbit IgG conjugated with Alexa488 (diluted 1:500; #4412; Thermo Fisher Scientific), fluorescence was detected using a CytoFLEX S flow cytometer (Beckman Coulter). The data were analyzed using FlowJo v10.8.1 software (FlowJo LLC).

**ExpLBM-derived chondrocyte sheet fabrication**

To induce chondrogenic differentiation in adherent culture conditions (two-dimensional chondrogenic induction, 2-DCI), ExpLBM cells (1 × 10^5^) were suspended in 500 μL of ExpLBM medium and were seeded on 24-well culture plates coated with 4 μg/mL of human plasma fibronectin (Merck). After 3–6 days of culture in ExpLBM medium, cells were washed with 1× PBS (−) and treated for 6 days with STEP1 medium (CDM2 medium + 3 μM of CHIR99021 + 10 ng/mL of FGF2 + 50 μg/mL of ascorbic acid + 1× ITS). STEP1 medium was then washed with 1× PBS (−) and then replaced with STEP 3 medium (CDM2 medium + 50 μg/mL of ascorbic acid + 30 ng/mL of bone morphogenetic protein 4 + 10 ng/mL of transforming growth factor beta 1 + 10 ng/mL of growth differentiation factor 5 + 1× ITS), and then cultured for 6–10 days. Culture media were replaced with fresh differentiation media every 3 days. For ExpLBM-derived chondrocyte sheet fabrication, ExpLBM-derived chondrocytes (1–2 × 10^7^) were seeded on temperature-responsive culture inserts (CellSeed Inc.) and cultured for 5–10 days with STEP3 medium containing 10 μM of Y27632 and 1% FBS. The culture media were replaced with fresh media every 3 days. ExpLBM-derived chondrocyte sheets were kept at 25°C for 30 minutes for detachment and were then manipulated and visually confirmed for strength and tearing, and then divided for each analysis.

**Real-time quantitative reverse-transcription-polymerase chain reaction (qRT-PCR):** The total RNA was extracted using the RNeasy kit (QIAGEN) and cDNA was synthesized using ReverTra Ace qPCR RT Master Mix with gDNA Remover (TOYOBO). Following that, with cycling conditions being as follows: denaturation at 95°C for 30 seconds, annealing at 62°C for 30 seconds, and elongation at 72°C for 30 seconds, cDNAs were used as templates for qRT-PCR analysis using gene-specific primers on the AriaMX real-time PCR system (Agilent Technologies). The gene expression level was calculated using the ΔΔCt method. The primer sequences were described as follows: *COL2A1*, forward (F) 5′-CCTGAGTGGAAGAGTGGAGACT-3′ and reverse (R) 5′-TCCTTGCTCTTGCTGCTCCA-3′; *ACAN*, F 5′-GGCACAGCCACCACCTACAA-3′ and R 5′‑AGCGACAAGAAGAGGACACCG-3′; *SOX9*, F 5′-AAGCTCTGGAGACTTCTGAACGA-3′ and R 5′-CGCCTTGAAGATGGCGTTGG-3′; *COL1A1*, F 5′‑CCACTGCAAGAACAGCGTGG-3′ and R 5′-GTGTGACTCGTGCAGCCATC-3′; *COL1A2*, F 5′-GGATGAGGAGACTGGCAACC-3′ and R 5′-TTGCCCTCAGCAACAAGTTC‑3′; *RUNX2*, F 5′-TCAACGATCTGAGATTTGTGGG-3′ and R 5′-GGGGAGGATTTGTGAAGACGG-3′; *MMP13*, F 5′-CATGAGTTCGGCCACTCCTT-3′ and R 5′-CCTGGACCATAGAGAGACTGGA-3′; *COL10A1*, F 5′‑CCCAGCACGCAGAATCCATC-3′ and R 5′-AGTGGGCCTTTTATGCCTGT-3′; *IHH*, F 5′-CGGTGGACATCACCACATCA-3′ and R 5′-CGTGGGCCTTTGACTCGTAA-3′; *TBP* (internal standard), F 5′-GAGCTGTGATGTGAAGTTTCC-3′ and R 5′-TCTGGGTTTGATCATTCTGTAG-3′.

**Immunohistochemistry:** Sheets or tissues were fixed in a 10% formalin neutral buffer solution (FUJIFILM Wako), and paraffin-embedded samples were sectioned (4-μm thickness). Tissue samples were deparaffinized, and antigens were activated by heating slides in 10 mM of citrate buffer (pH 6.0). After treating the sections with 0.3% H_2_O_2_/MeOH, the samples, treated with primary antibody (diluted 1:200) overnight at 4°C, were incubated with a blocking solution [(3% NGS)/0.1% Triton X-100/1× PBS (−)], followed by secondary antibody (diluted 1:400) for 1 hour at room temperature. The antibodies were diluted with the blocking solution. Samples were embedded with Fluoromount-G (SouthernBiottech) after staining with DAPI and the images were acquired using the BZ-X710 camera (Keyence). The antibodies used were as follows: anti-hVIMENTIN (catalog no. 10515; Progen Biotechnik), PRRX1 (catalog no. ZRB2165; MilliporeSigma), SOX9 (catalog no. AB5535; Merck), COL2 (catalog no. MA1-37493; Thermo Fisher Scientific), COL1 (catalog no. 1441-01; SouthernBiotech), RUNX2 (catalog no. 12556; Cell Signaling Technology), and ACAN (catalog no. 13880-1-AP; Proteintech).

**ExpLBM-derived chondrocyte sheet transplantation:** In the transplantation experiments, 6- to 10-week-old X-linked severe combined immunodeficiency (X-SCID) rats (F344-*Il2rg^em1Iexas^;* The Institute of Medical Science, The University of Tokyo) were used (each sheet transplantation; independent experiments n=3, total six animals, minimum number of animals that is reasonable). The experiments using rats and animal care procedures were approved by the Okayama University Animal Care and Use Committee (2020814). Two animals were housed in each cage, with daily standard chow and unlimited access to water. The rats were anesthetized using isoflurane by inhalation anesthesia machine (4-5% induction, 2-3% maintenance). A medial parapatellar incision was made on the knee. It was done randomly operation that was no left-right difference. Using a biopsy punch (Kai Industries), the patella was moved laterally and an osteochondral defect (1-mm drill hole × 3 defects; 1-mm depth) was created in the patellofemoral groove. Marrow bleeding was observed during the formation of the osteochondral defect, and the rats were sacrificed by the carbon dioxide gas method four weeks after ExpLBM-derived chondrocyte sheet transplantation, where following the sacrificing, tissue samples that were fixed for one week in a 10% formalin neutral buffer solution (FUJIFILM Wako) were paraffin‑embedded at Okayama University Medical School’s Central Research Laboratory. The engraftment of human-derived cells into rat knee cartilage defects is assessed by staining for hVIMENTIN.

**Statistical analysis:** The data were analyzed using the Prism 9 software. All data from three independent experiments are presented as the mean ± standard error of the median, and statistical significance was determined using a two-tailed t-test and the Bonferroni method.
